# Computational Neuroscience’s Influence on Autism Neuro-Transmission Research: Mapping Serotonin, Dopamine, GABA, and Glutamate

**DOI:** 10.3390/biomedicines13061420

**Published:** 2025-06-10

**Authors:** Victoria Bamicha, Pantelis Pergantis, Charalabos Skianis, Athanasios Drigas

**Affiliations:** 1 Net Media Lab & Mind & Brain R&D, Institute of Informatics & Telecommunications, National Centre of Scientific Research ‘Demokritos’, 153 41 Agia Paraskevi, Greece; 2 Department of Information & Communication Systems Engineering, University of the Aegean, 832 00 Karlovasi, Greece; cskianis@aegean.gr

**Keywords:** computational neuroscience, computational psychiatry, autism spectrum disorder, neurotransmitter function, serotonergic system, dopaminergic system, glutamatergic system, GABAergic system, computational modeling, machine learning

## Abstract

Autism spectrum disorder is a complex and diverse neurobiological condition. Understanding the mechanisms and causes of the disorder requires an in-depth study and modeling of the immune, mitochondrial, and neurological systems. Computational neuroscience enhances psychiatric science by employing machine learning techniques on neural networks, combining data on brain activity with the pathophysiological and biological characteristics of psychiatric–neurobiological disorders. The research explores the integration of neurotransmitter activity into computational models and their potential roles in diagnosing and treating autism using computational methods. This research employs a narrative review that focuses on four neurotransmitter systems directly related to the manifestation of autism, specifically the following neurotransmitters: serotonin, dopamine, glutamate, and gamma-aminobutyric acid (GABA). This study reveals that computational neuroscience advances autism diagnosis and treatment by identifying genetic factors and improving the efficiency of diagnosis. Neurotransmitters play a crucial role in the function of brain cells, enhancing synaptic conduction and signal transmission. However, the interaction of chemical compounds with genetic factors and network alterations influences the pathophysiology of autism. This study integrates the investigation of computational approaches in four neurotransmitter systems associated with ASD. It improves our understanding of the disorder and provides insights that could stimulate further research, thereby contributing to the development of effective treatments.

## 1. Introduction

In the 1980s, cognitive science advanced with symbolic cognitive architectures and neural networks using human behavioral data. However, computer hardware and machine learning were not advanced enough to simulate complex mental processes. Cognitive neuroscience emerged, mapping the information processing modules of cognitive psychology to the brain, but without computational rigor [1].

Understanding how the brain functions is a challenge for various scientific fields. Simple behaviors emerge from the complex interactions among numerous neurons in different brain regions [2]. Computer hardware and software innovations have enabled the modeling of the mind and brain, highlighting the transformative potential of artificial intelligence. Consequently, this has allowed fields like cognitive science, computational neuroscience, and artificial intelligence to explain human cognition using neurobiological computational models [1].

Computational neuroscience uses quantitative models to describe neurons, circuits, and brain regions. These models are based on biophysical foundations, such as current balance and ion conductance. Two scientific discoveries in the last century, the creation of the action potential by Hodgkin and Huxley [3], which is related to the mechanism of information transmission from cell to cell, and the “cable theory” of Wilfrid Rall [4], which refers to the description of signal transmission along neurites, were decisive for the creation of neuronal modeling. The effectiveness of these approaches is enhanced by an understanding of computer hardware and ion channel dynamics [5].

Computational models are crucial to studying the nervous system, providing various approaches to understanding phenomena. Descriptive models concisely relate experimental data, while mechanistic models focus on the system’s function based on anatomy and physiology. Interpretive models use computational and mathematical principles to explore the function of the nervous system. Finally, fitted models require explicit mathematical relationships to explain observations. These models rely on experimental hypotheses to predict possible mechanisms underlying human neural diseases [6].

The prevalence of autism spectrum disorder (ASD) has increased significantly in the last decade, probably due to the advances in genetic research, early recognition of autistic traits, and new guidelines and diagnostic criteria [7]. Epigenetic modifications, prenatal exposure, immune dysfunctions, and alterations in the gut microbiome contribute significantly to the onset and progression of the disease [8,9,10]. The biology of autism is challenging due to its multiple etiologies, including gastrointestinal diseases, immune system disorders [11], and early signs, like the use of gaze, the way of communication, and the use of sounds and words [12]. It complicates modeling and can confound studies of autism, as distinct subgroups are not identified solely by their behavioral characteristics [13].

The research indicates that several psychiatric, neurological, and neurodegenerative disorders result from alterations in the synapses of the brain’s neuronal network, affecting the transmission of neuronal messages in cortical and subcortical areas [14]. ASD cases show common symptoms, suggesting shared neurodevelopmental deficits. Neurotransmitter dysfunctions are implicated in the pathophysiology of ASD, as they play a crucial role in brain development, synaptogenesis, memory, behavior, and gastrointestinal functions [8]. Neurotransmission mechanisms span multiple research areas and require an interdisciplinary approach. Enhanced computing platforms have furthered the mathematical tools utilized to study neurobiological processes. Simulating neuronal functions requires computational models with strong assumptions that accurately reflect synaptic and neuronal mechanisms and their parameters [15].

This study’s findings indicate that computational models in psychiatry and neuroscience can examine how initial dysfunction creates progressive disorders through neural development and plasticity. These models can characterize individual differences, tailor biobehavioral treatments, and predict behavioral outcomes at the personal level. As technology improves, these approaches can help create personalized interventions for individuals with developmental disorders, such as ASD [16].

Computational models are increasingly used to understand neuronal function, particularly the action of specific neurons and neural networks. The models are based on real neuron morphologies and can be combined to create biophysical models of large-scale neuronal circuits. An illustrative example is NEURON simulation software that standardizes computational neuroscience methodology, facilitating the reproduction of simulation results and allowing users to define ion channels and their distribution along neurites [5].

Recent neuroimaging techniques, such as positron emission tomography (PET) and single-photon emission computed tomography (SPECT), combined with magnetic resonance imaging (MRI), have been utilized to explore the neural basis of autism spectrum disorders (ASDs). These methods reveal alterations in the serotonergic, dopaminergic, glutamatergic, and GABAergic systems that are associated with ASD, helping to inform the development of effective treatments for autism [17]. Maintaining the complex balance of excitation and inhibition between nerve cells is crucial to the proper function and homeostasis of the central nervous system [18].

However, computational tools have limitations due to their technical expertise and uncertainty. To achieve a meaningful interaction between clinicians, experimentalists, and theorists, focusing on usability, promoting open source communication, and popularizing databases would help improve therapeutic methods [19].

The researchers and medical professionals are becoming concerned about the rising incidence of autism. Its multidimensional etiology, including its alterations in neurotransmission and the chemical signaling between neurons, makes modeling and the research challenging.

The essential role of neurotransmission in the pathophysiology of autism has spurred this study. More specifically, this research explores the possibilities of incorporating data on neurotransmitter functions into computational models, aiming to provide insights into the brain’s chemical action and new biological clues in diagnosing and treating autism. This study investigates computational approaches used in four primary neurotransmitter systems, where the following deficiencies are associated with ASD: serotonergic, dopaminergic, glutamatergic, and GABAergic systems. It contributes to the research process, by providing information from various neurotransmitter systems that play a significant role in brain function, enhancing the neurobiological understanding of the disorder. Additionally, a concise report on using computational methods in diagnosing and treating autism may aid in developing targeted interventions.

This review was implemented in light of a narrative review, allowing the combination of previous studies to enhance our knowledge and understanding. At the same time, it involves the interpretation and criticism of data, providing a multifaceted and flexible approach to this research topic [20,21,22]. We searched bibliographic sources using the following search terms: computational neuroscience, computational psychiatry, autism spectrum disorder, neurotransmitter function, serotonergic system, dopaminergic system, glutamatergic system, GABAergic system, computational modeling, and machine learning.

This study consists of nine sections. The first section is the introduction, and the second outlines the theoretical background. The third through seventh sections present this study’s findings. The eighth section discusses the implications of the findings, while the ninth section summarizes the conclusions.

## 2. Theoretical Knowledge

### 2.1. Computational Neuroscience

Recent advancements in cognitive science, computational neuroscience, and artificial intelligence suggest that an understanding of the brain can be achieved through computational models [1,23]. Cognitive science decomposes complex cognitive processes into computational components, while computational neuroscience demonstrates dynamic interactions between neurons implementing computational functions [1].

Computational neuroscience creates a mechanistic brain model, capturing human behavior through reinforcement learning and game theory models [24]. It focuses on modeling brain processes to understand information processing, discover new phenomena, and simulate experiments, aiming to study and interpret electrical and chemical signals in the brain [2].

Computational and mathematical models are valuable tools in computational neuroscience for investigating and understanding neural computation and the interconnections within neural systems. There are three ways to use these models: Computational Level: This level explores the “what” and “why” of the brain’s computations. Algorithmic Level: Here, the focus is on determining “which” algorithmic procedures can effectively represent these physical computations. Physical Level: This level examines “how” these procedures apply in practice. Each model addresses specific scientific questions, concentrating on the primary variable of interest [25].

Three types of models that provide computational insights from brain activity data are connectivity, decoding, and representation models [1]. Moreover, data-driven models simulate brain activity, while task-driven models predict behavior, bridging the gap between neuronal activity and cognitive functions [2].

More specifically, computational neuroscience utilizes various models, including neural network models, deep neural networks, spike neural networks, convolutional neural networks, recurrent neural networks, reinforcement learning models, biophysically detailed models, Bayesian models, and connectionist or parallel distributed processing (PDP) models [1,5,14,19,24,26,27,28,29]. These models enhance our understanding of biological neural networks and computational functions, enabling parallel distributed processing [1]. Biophysically detailed models can analyze many aspects of neuronal excitability involving elements and variants of distinct genes [5]. Deep neural networks aim to achieve behavioral relevance and neuronal selectivity, while Spiking Neural Networks (SNNs) offer energy efficiency [24,27]. Convolutional neural networks (CNNs) are second-generation deep neural networks inspired by the ventral visual stream of the brain [28]. Genetic models are also used to determine phenotypes, providing a deeper understanding of the underlying mechanisms that shape phenotypes [18].

**Figure 1:**
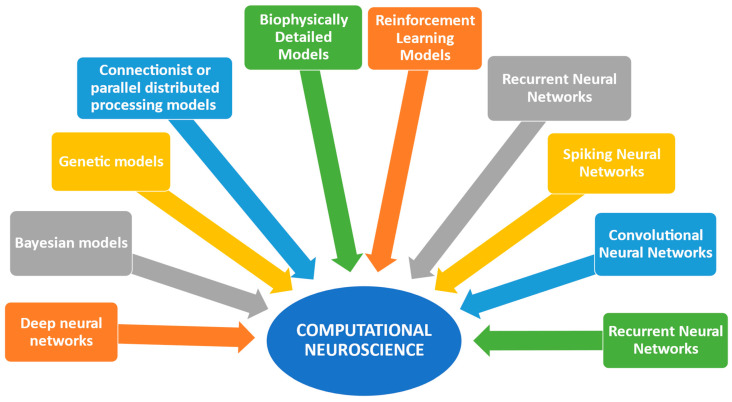
Computational methods and artificial intelligence tools improve the effectiveness of computational science.

Various studies [1,5,6,14,15,16,19,24,27,28,29] indicate that computational neuroscience combines computational models and artificial intelligence methods to comprehend human brain function and behavior (Figure 1).

### 2.2. Neurotransmitters

The nervous system regulates behavior through neurotransmitters, chemical substances released by neurons at synapses. These substances communicate information through synaptic transmission, enhancing or inhibiting nerve transmission. They are stored in vesicles at neuron terminals before release [30,31,32].

Monitoring neurotransmitter levels is crucial for understanding and treating mental illnesses. Neurotransmitters are analyzed using conventional methods, such as brain microdialysis, high-pressure liquid chromatography (HPLC), mass spectroscopy (MS), capillary electrophoresis (CE), electroencephalography (EEG), proton nuclear magnetic resonance, and magnetic resonance imaging (MRI). The research reports that nanoparticles and electrochemical analysis offer sensitivity, lower concentration detection, and faster response times, allowing for the real-time monitoring of neurotransmitter dynamics [33].

Based on their functions, there are three categories of neurotransmitters: excitatory neurotransmitters, which stimulate action in target cells; inhibitory neurotransmitters, which prevent action by blocking the receiving neuron; and modulatory neurotransmitters, which affect the activity of other neurotransmitters by sending signals to many neurons simultaneously [34].

**Serotonin (5-HT)** is a neurotransmitter produced in the central nervous system by neurons of the caudal midbrain in the dorsal and median raphe nuclei [35]. Its inhibitory effects are associated with various behaviors, such as feeding, body weight regulation, aggression, obsessive compulsive disorder, alcoholism, anxiety, emotional disorders, motor system function, sleep cycles, circadian rhythms, respiratory stability, and reward processing [36]. In addition, serotonin plays a critical role in neuronal development, plasticity, cortical synaptic plasticity, somatosensory cortex morphogenesis, and patterned cortical glutamatergic connections [37]. The multifunctional action potential of the brain’s serotonin system is due to various receptors, with recent studies showing their involvement in behaviors and molecular mechanisms related to autism in the development of the autistic phenotype, such as stereotypical behavior and intense anxiety, deficits in social interactions, memory, and learning [38,39]. Moreover, 5-HT modulates glutamate- and GABA-mediated transmission in the central nervous system, causing various effects [40].

**Dopamine (DA)** is a neurotransmitter that belongs to the catecholamine family. It plays a significant role in modifying behavior and links to reward mechanisms and social motivations, which are crucial in understanding social deficits in individuals with autism spectrum disorder (ASD). An overactive dopaminergic system in the orbitofrontal–limbic circuit can result in poor emotional regulation, often leading to impulsive and aggressive behaviors in those with ASD. Conversely, deficiencies in prefrontal dopamine can cause cognitive impairments in these individuals [17]. Specifically, the dopaminergic system in the brain has four main pathways: the Nigro-Stiatal, the Μesolimbic, the Μesocortical, and Tuberoinfundibular, the altered functions of which lead to Parkinson’s disease and to the disruption of the emotional and reward systems, cognitive and emotional behavior, and to the inhibition of prolactin release [41].

**Glutamic acid (Glu)** is an excitatory amino acid neurotransmitter in the central nervous system, particularly enhancing memory function. It is synthesized from glutamine, a nutrient in various foods, such as meat, eggs, and dairy products. It is the most abundant excitatory neurotransmitter in the brain, allowing the excitation of almost all synapses [31].

**Gamma-aminobutyric acid (GABA)** is the brain’s main inhibitory neurotransmitter, essential for many crucial processes. Its influence extends to proliferation, migration, synaptic maturation, differentiation, and programmed cell death. However, disturbances within the GABAergic system have been linked to autistic behaviors, shedding light on challenges in information processing and the delicate development of social skills [42,43,44]. Understanding GABA’s role may unlock new insights into these complex conditions, enhancing our approach to fostering social connectivity and understanding [45].

Glutamate enters astrocytes, converts to glutamine, and is transported to neurons for glutamate and GABA production. Changes in one of the neurotransmitters can affect the other [46]. Efficient brain function relies on the balance between glutamatergic and GABAergic neurotransmission [41,47]. Alterations in these systems, such as high levels of glutamate or low levels of GABA, may contribute to the pathophysiology of ASD, affecting neuronal network organization and information processing. Seizures, hypersensitivity, social deficits, repetitive behaviors, sensory, memory, emotional, oxidative stress, reduced social competence, self-stimulatory behavior, seizures, and anxiety disorders are associated with this condition [8,47,48,49].

**Figure 2:**
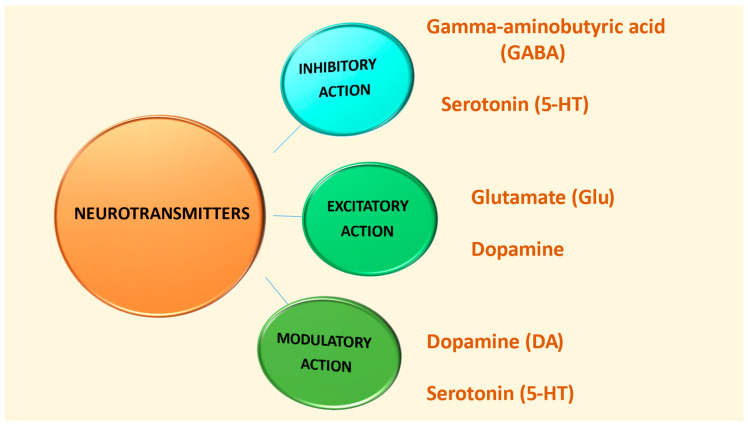
Sorting GABA, serotonin, glutamate, and dopamine based on their roles.

Figure 2 and Table 1 depict the classification of the neurotransmitters GABA, serotonin, glutamate, and dopamine according to their action [10,30,31,32,34,45].

### 2.3. Autism

Autism spectrum disorders (ASDs) are a neurodevelopmental disorder characterized by deficits in social–emotional and cognitive domains. It is diagnosed early in life, with 1 in 54 children diagnosed by the age of 8 years. The precise neurobiological basis is unknown due to heterogeneous behavior and divergent genetic, transcriptional, and epigenomic characteristics. Different brain regions contribute to distinct aspects of ASD symptomatology, including the cortex, amygdala, cerebellum, and basal ganglia [50,51].

Possible causes of autism include changes in detoxification systems, toxin accumulation, immune system dysfunction, gastrointestinal system dysfunction, and brain damage. However, the combination of these elements does not confirm the cause. In addition, stress disrupts metabolic pathways and can cause gut dysfunction, immune system activation, mitochondrial dysfunction, and oxidative stress. Since the total number of possible interactions in autism is high, a systemic approach to the condition is necessary [13].

Furthermore, the etiology of autism involves environmental contamination [52]. Tartaglione et al. included different methods for assessing prenatal and postnatal exposure to pollutants in their study, from measurements in biological panels (e.g., blood, urine, and hair) to computational models based on geographic information systems. Their findings indicate that exposure to environmental pollutants, such as metals (e.g., lead, mercury, copper, and chromium), pesticides, and air pollutants (carbon monoxide and dioxide) [53] during early life, is a risk factor for neurodevelopmental disorders (NDDs), including ASD [52].

Symptoms of ASD appear during the first three years of life when the rapid formation and maturation of brain synapses occur [8]. Brain development processes such as synaptogenesis, arborization, migration, synaptic pruning, and plasticity aim to create a functional brain [54,55]. Neurotransmitters and their receptors play a critical role during development, with Glu receptors being vulnerable to changes in neurotransmission, leading to neurological and psychiatric disorders [8].

Impaired social interactions and altered sensory perception are hallmarks of autism, potentially influencing social behaviors [56]. The research indicates that early multimodal stimulation may alleviate deficits [57,58,59,60] and systematic developmental studies can contribute to sensory processing [58].

Autism spectrum disorders are highly heterogeneous neurodevelopmental conditions. Computational models are essential for comparing and categorizing the effects of ASD on synaptic function, considering environmental and genetic factors and their unique mechanisms and consequences [61].

**Figure 3:**
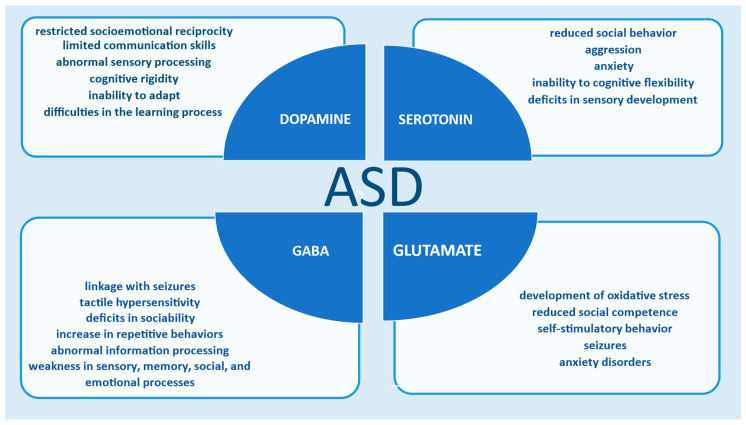
Neurotransmission is connected to the emergence of ASD symptoms.

Several studies [10,17,30,31,32,34,38,47,56] indicate a connection between neurotransmitters and autism. The functionality of GABA, serotonin, glutamate, and dopamine is associated with the development of ASD symptoms. Changes in the synthesis and release of these chemical messengers affect brain function and the overall development of autism (Figure 3).

## 3. The Computational Neuroscience Approach to Understanding Neurotransmitter Function in ASD

The study of cognitive brain function has created the fundamental framework for artificial intelligence (AI) development. Digital technology has significantly advanced artificial systems by replicating the physiological activities of the human brain. However, these systems cannot simulate human psychological changes [62].

AI, influenced by neuroscience and cognitive psychology, enhances the decoding of neural activity, allowing for a better understanding and prediction of human behavior. As such, it is a powerful tool for neuroscientists and clinicians at the diagnostic and therapeutic levels [63]. Neural network integration that “learns to make inferences” with complex model-building learning mechanisms helps interpret how the human mind swiftly and effectively sees and comprehends the universe [64].

Artificial intelligence and neuroscience cooperate to understand brain mechanisms and advance human cognition, enabling large-scale simulations of neural processes. This interaction has led to the design of artificial neural networks (ANNs) and applications, pointing to a common goal of identifying and diagnosing neurological disorders [28].

The use of diagnostic criteria in clinical manuals leads to the identification of psychiatric and developmental disorders, which frequently have their diagnoses based on self-report assessments and behaviors. Advances in computer and neuroimaging datasets have allowed the researchers to use artificial intelligence to identify, model, and potentially treat these disorders [63].

Neural simulations, which bridge molecular and behavioral levels, help to elucidate the causes of behavior in ASD and connect network dynamics with molecular and genetic properties, thereby requiring high computational power [65]. Many co-expressed ASD susceptibility genes are linked to synaptic plasticity, neuronal development, and differentiation, underscoring the limitations in neural circuit development as factors under investigation [66].

Krishnan et al. [67] employed a machine learning approach to predict autism-related genes. They developed a statistical model that captured the connectivity patterns of known autism-related genes within the brain network. A Bayesian approach was utilized to construct the functional brain network. The model predicted whether “unknown/unlabeled” genes in the network resembled autism-related genes. This computational method enables genome-wide ASD gene discovery by ranking all genes in the human genome according to their potential pathogenic involvement in ASD. Furthermore, data analysis uncovered spatiotemporal patterns and identified hundreds of novel genes in previously connected brain regions, such as the cerebellum and striatum [67].

A few years later, the largest exome sequencing study on autism spectrum disorder identified 102 genes associated with ASD using a Bayesian-enhanced analytical framework. The study examined the cellular function of ASD-associated genes in the developing human cortex, revealing their enrichment in maturing excitatory and inhibitory neurons, their role in neuronal communication, and their separable functions, contributing to the our understanding of the neurobiology of ASD [68].

An earlier genetic study used the sequencing of protein-coding regions of the genome to detect single-nucleotide variants in children with autism spectrum disorder. These variants are more prevalent than expected and may disrupt the genes. The genes are particularly abundant in proteins involved in brain function and gene regulation. The researchers utilized computational models to identify enriched co-expression of ASD risk genes in specific cortical regions from early to mid-gestation [69].

Neural and artificial computational systems exhibit various levels of mechanistic organization, including large systems such as the brain and cerebellum, which are decomposed into subsystems, like the cortex and brainstem, and further subdivided into smaller units of neural structures. Computational neuroscience examines these levels, aiming to uncover the individual properties of the system and contribute to the understanding of neural activity at multiple levels [70].

More specifically, computational neuroscience aims to understand how the nervous system processes information for cognitive function and adaptive behaviors. It employs mathematical and computational models to map sensory input to neural responses thereby explaining representational processes, neuronal dynamics, and brain control. Since the brain functions as a deep recurrent neural network, computational neuroscience will increasingly rely on complex models [27]. Additionally, it leverages expertise in developing simulation software, particularly for multiscale modeling. It began with the Rall wire equation and Hodgkin–Huxley models, which have now been extended to molecules and large neural networks. Simulators such as GENESIS support detailed simulations of biochemical pathways within neurons and neural network models [71]. The Vladusich study [72] suggests that modifying existing computational models of healthy brain function could help us understand how neurotransmitter imbalances induce the behavioral symptoms associated with autism.

However, variability in connectivity and synaptic properties between experiments hinders the development of accurate computational models. Standardized experimental methods and large-scale data collection are required to characterize connections between different cell types. The rate of connectivity and the characteristics of synaptic signals are related to the set of properties of the communicating neurons, providing important information about the neuronal system [73,74].

The brain’s computational power in memory and learning is enabled by converting electrical signals into chemical neurotransmitter information at synapses. As synapses become more complex, computational and hardware tools must evolve to evaluate these models. The primary hub for information transfer in brain circuits is the synapse, which is controlled through several different time-scale synaptic plasticity mechanisms. Synaptic function and plasticity depend on the types of neurons and molecular mechanisms. Understanding information processing in synaptic networks requires computational models and real-time analytical measurements [61].

Robertson and Baron-Cohen [75] highlight the “normal microcircuit view”, which suggests that autistic traits at different levels share common neural mechanisms. Furthermore, sensory symptoms in autism are a key feature of neurobiology, affecting social and cognitive functioning in adults. These symptoms are visible early in development and predict diagnostic status later in childhood. They reflect changes in neural circuitry related to the sensory system. They emphasize the link to the imbalance of excitatory and inhibitory signaling, such as reduced inhibition relative to excitation in autistic visual circuits, and alterations in the GABAergic system, constituting key disease biomarkers.

The study by Thomas and Johnson [76] used a computational approach to link excitatory–inhibitory synaptic imbalance and abnormal levels of synaptic strength to attentional deficits in autism. A network of excitatory and inhibitory conduction neurons of the Hodgkin–Huxley type formed the model. By modeling two competing microcolumns in the inferior temporal cortex (IT) and adjusting for synaptic strength levels, the results suggested that these imbalances could be responsible for some attentional deficits in individuals with autism.

Biophysical modeling and numerical simulation studies can accurately test hypotheses and quantify synaptic transmission in diseases such as autism that involve synaptic changes. Freche et al. [77] investigated the impact of a mutation in the SHANK3 protein on synaptic transmission in autism spectrum disorders, revealing that this mutation reduces synaptic currents in mature neurons. It is worth mentioning that Shank3 is an excitatory postsynaptic protein that regulates synaptic function in various brain regions. Furthermore, it is associated with increased stereotypic behavior and reduced sociability [78,79].

The research on neurotransmission has advanced significantly, using 3D morphological reconstructions, immunochemistry, calcium imaging, proteomics, and genomics. It has led to the creation of atlases such as the Allen Brain Atlas, which contains information on brain neurons, circuits, and the distribution of synaptic receptors in rodents and humans [80]. More precisely, the Allen Brain Atlas is an online resource that provides excellent neuroanatomy knowledge and data, making it a valuable tool for learning about the brain [81]. Computational models can leverage this dataset to build knowledge related to neurotransmission [80].

Computational approaches to promote mental wellness face numerous challenges, such as noise, incomplete and sparse data, validation, small sample sizes, reproducibility, reliability and validity, temporal dynamics, and generalizability. Noise in the data can impact the quality and reliability of the model, while a lack of data introduces biases. Validation is crucial for achieving accurate results, especially in environments where hyperparameters are optimized. Small sample sizes can lead to non-reproducible results and reduced statistical power. Reliability and validity are vital for producing generalizable results. Temporal dynamics, which may be disorder-related or independent, require repeated longitudinal assessments to model mental health. Clinical success depends on generalization, which can be challenging when sample data significantly deviate from clinical reality [29].

**Figure 4:**
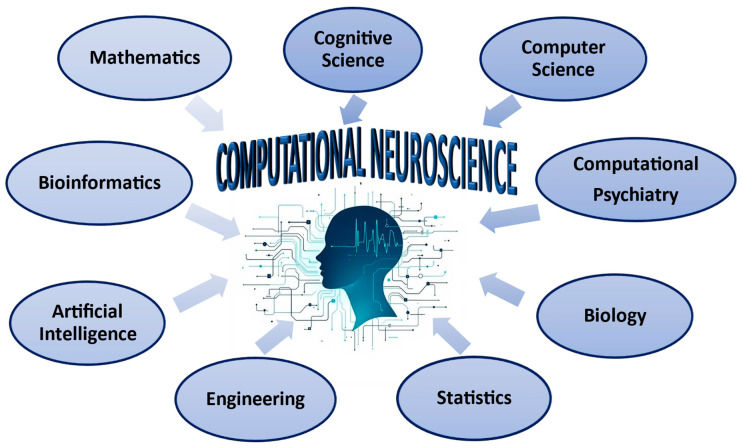
Computational neuroscience interacts with other scientific disciplines.

The research’s conclusions [1,5,6,27,63,64,65,67,69,70,73,74] highlight the necessary collaboration and acquisition of knowledge in Computational Neuroscience from different intellectual domains (Figure 4).

## 4. Serotonergic System in ASD and the Use of Computational Methods

Serotonin (5-HT) is a neurotransmitter in the brain and a hormone in peripheral tissues. It forms numerous terminals in brain structures and plays a crucial role in regulating behavior, social interactions, stereotyped behavior, anxiety, stress, learning, and memory, which are the main symptoms of ASD [38].

In addition, a large body of research on the neurochemical correlates of autism has provided evidence for the role of serotonin in alterations in neurogenesis, brain development, sensory perception, motor function, and sleep and its association with social deficits, aggression, and hyperactivity [82]. Its receptors, classified into seven subfamilies, contribute crucially to neurite outgrowth, dendritic spine morphology, and neuronal circuit formation. 5-HT systems are associated with several diseases, making them a critical class of biogenic amines. Recent studies have focused on 5-HTR modulators as ASD therapeutics [83].

Understanding the mechanisms of serotonergic signaling is challenging because of four levels: genomic, biochemical, electrophysiological, and behavioral. Changes at each level affect the other three, making interpretation difficult. Mathematical models can help study the simultaneous effects of interacting levels and evaluate hypotheses through computer simulations. Best et al. [36] developed a mathematical model that explains how serotonin is synthesized, released, and reabsorbed in a single neuron terminal, considering the effects of autoreceptors, tryptophan transport, serotonin metabolism, and firing-rate dependence. The findings revealed that serotonergic systems maintain homeostasis despite biological fluctuations through various homeostatic mechanisms, including serotonin transporters and autoreceptors, ensuring a robust response to biological signals. Autoreceptors play an important role in regulating 5-HT chemistry. They reduce the impact of changes in the firing rate and polymorphisms in extracellular 5-HT.

ASD is associated with gastrointestinal problems, with elevated blood serotonin levels being a biomarker associated with autism. Specifically, one-quarter to one-third of individuals with ASD have hyperserotonemia. Platelets, which do not produce serotonin, utilize a serotonin reuptake transporter (SERT) for its uptake from the gut, a process linked to ASD. Kareva [84] in her research introduces a mathematical model to study the interactions between gut serotonin and its effect on the enteric nervous system (ENS). The study’s computational model identifies three key factors influencing the ENS: serotonin production, clearance, and growth factor function. To illustrate how these parameters might relate to autism spectrum disorder, the model scales the findings from studies conducted on mice to humans. Essentially, running simulations that alter all three of these factors revealed that they can be associated with the development of ASD symptoms.

Regional serotonergic fiber-density changes have been linked to several mental illnesses, including autism spectrum disorder [85]. A different study using supercomputer simulations has shown that the self-organization of the brain matrix of serotonergic axons is a critical issue in neuroscience. The study compared the regional densities of these fibers with the actual fiber densities in the mouse brain, revealing strong qualitative similarities in the forebrain and midbrain. The study suggests that brain geometry can significantly influence the density of serotonergic fibers, influencing brain development, plasticity, and evolution [86].

Modern medical imaging technologies enhance image information, necessitating extensive analysis by doctors [87]. However, images, including those of the brain, often exhibit low contrast, tissue variability, and fuzziness, leading to uncertainty in diagnosis and prediction [88]. Fuzziness in brain images presents a challenge in distinguishing between the image and the background, complicating segmentation due to various factors. The FCM (Fuzzy C-Means) algorithm based on the fuzzy theory addresses this uncertainty and imprecision, providing numerous advantages. It can help to better understand and describe features in brain images. In particular, it allows for the analysis of overall features when segmenting brain images and extracting detailed information. In addition, it reduces varying degrees of noise, weak boundaries, and artifacts in brain images [89]. Another effective neuroimaging method is PET [17]. PET probes with good brain penetration, high binding affinity, specificity, metabolic stability, and high volume of distribution can provide information about neurotransmitter function in autism, including serotonin [90].

Machine learning algorithms are used as classification methods to predict ASD, thereby enhancing the developmental trajectory of a child’s mental health [91]. Machine learning classifiers are increasingly used to analyze functional magnetic resonance imaging (fMRI) data, extracting new information from neuroimaging data. Classifiers are functions that take values of independent variables and predict the dependent variable, allowing for more detailed analysis and interpretation [92].

Itahashi et al. [93] developed a classifier for adults with ASD using a large-scale resting-state fMRI dataset of 730 Japanese individuals. The study aimed to extract evidence regarding the pathophysiology of the disease at the functional network level, neurotransmitters, and clinical symptoms associated with the brain of individuals with autism. The adult ASD classifier, which was generalized to adults in the USA, Belgium, and Japan, was transferable to children and adolescents. It identified 141 functional connections related to difficulties in social interaction and serotonin and dopamine levels. The classifier also mapped ADHD, schizophrenia, and major depressive disorder onto the biological axis, providing a generalizable and transferable tool for assessing related illnesses.

## 5. Dopaminergic System in ASD and the Use of Computational Methods

Autism-related social deficits and stereotypic behaviors are attributed to altered dopaminergic signaling in the midbrain, with neuroreceptor imaging providing potential evidence for dopamine dysfunction [94].

Dysregulated dopamine signaling in brain regions associated with autism spectrum disorder could affect brain activity in various brain structures. The dopamine hypothesis suggests that functional dysregulation of DA projection pathways could contribute to behavioral changes, such as reduced reward value, abnormal sensory processing, and repetitive movements. It indicates that hyper- or hypo-dopaminergic signaling could exacerbate behavioral changes associated with ASD [50].

Neurons’ functional characteristics can influence the patterns of structural connectivity within the nervous system, while synaptic plasticity affects the structures of neuronal networks. Neuromodulators play a crucial role in distinguishing the mechanisms of synaptic transmission and integration, acting as regulators of neuronal excitability by influencing the release of neurotransmitters [95]. Béroule study [96] reports that the guided propagation memory model can help investigate the impact of neuromodulation lesions in developmental disorders such as autism by controlling the expression of genes involved in neurotransmitter metabolism. Specifically, monoaminergic imbalance in autistic disorders is altered by allelic variants of gene promoters involved in monoamine transport and metabolism. Furthermore, the interaction between neurotransmitters, that control brain functions, influences the severity of the impairments.

Computational neuroscience models focus on dopaminergic modulation, a neuromodulator crucial for motor control, directed behavior, motivation, reinforcement learning, and cognitive functions, such as working memory, planning, attention, and time perception. This highlights its importance in learning and cognitive abilities [97]. Experiments investigating how the dopamine system affects reward choices are influenced by reinforcement learning, which may help us understand other neuromodulatory systems in the brain. Neuromodulators like dopamine play a critical role in cognitive disorders. By combining the biological findings with reinforcement learning concepts, these models describe algorithms in the brain for evaluating value and making decisions, creating new perspectives in the research [98]. In particular, reinforcement learning (RL) models, such as Temporal-Difference RL (TDRL) and Temporal-Difference STDP, explain the role of DA modulation in learning tasks. These models assign values to specific actions, which can change due to reward prediction errors associated with DA. However, each model has limitations in examining the modulatory influence of DA on selected choices [95].

Neural network models use simplified neuronal units and dynamics to understand how circuit interactions and the neurochemical actions of dopamine and other neurotransmitters support and are linked to cognitive processes, such as action selection, learning, and working memory [99]. The research suggests that the basal ganglia play a critical role in dopaminergic gating, selectively updating goal representations in the prefrontal cortex (PFC) through recurrent pathways and learning of hierarchical tasks through computational models [98].

The cross-task (XT) computational generalization model captures the functional segregation of the prefrontal cortex in ASD. It suggests that the prefrontal cortex is central to cognitive control, while the mesolimbic dopamine system mediates the flexible adaptation of control. This model has been used to study individuals with frontal damage and healthy controls. Kriete and Noelle [100] report that the functional segregation and mechanisms of the XT model can explain the executive processing profile of individuals with autism. The model suggests the mechanistic application of control and flexibility separately can describe unique patterns of executive performance. Their study reveals that the attenuation of the effect of DA on pyramidal cells in the prefrontal cortex can explain autistic performance, possibly due to PFC/DA interactions in this specific executive processing profile.

Neural systems models can simulate dopamine effects at various levels of abstraction. Biophysical models based on Hodgkin–Huxley conductances and compartmentalized morphological structures can directly apply DA effects. Abstract models, such as connectionist models, start from specific assumptions about the pure effects of DA. Both approaches can complement each other; however, biophysical models are less suitable for investigating complex cognitive tasks. Therefore, studies at a higher level of model abstraction may be more appropriate [97]. A few years later, Cartier et al. [101] highlighted the importance of syntaxin 1 (STX1), a presynaptic protein that regulates synaptic vesicle fusion and the function of neurotransmitter transporters, including the dopamine transporter (DAT). In particular, STX1 regulates DAT functions and DA neurotransmission, affecting dopaminergic synapses [102]. DAT regulates DA signaling in the striatum, with synaptic release playing a critical role [103]. The researchers employed advanced animal models and biophysical methods to investigate the role of identified gene variants in the neurotransmission impairments observed in ASD. The study results identified two autism-associated variants in syntaxin and the dopamine transporter that affect dopamine reverse transport. This suggests the dysregulation of dopamine neurotransmission as a potential complication of autism spectrum disorder. Variants in the genes encoding STX1 and DAT alter DAT function, inhibiting DA reverse transport and associated behaviors [104].

The researchers are using noninvasive neuroimaging techniques in humans to investigate the neural correlates of reward prediction errors related to the role of dopamine in the neural network. In particular, when using functional magnetic resonance imaging (fMRI), they apply computational reinforcement learning models that generate prediction errors for each trial. As a result, they identify brain regions with significant correlates of dopaminergic activity in the striatum and frontal cortex [99].

According to the research, computational neuroimaging neuromodulation detectors are crucial for stratifying patients with spectrum disorders. In ASD, the dysregulation of neuromodulation mechanisms can cause abnormal prediction errors and behavioral consequences. Previous studies using single-photon emission computed tomography and positron emission tomography have reported group-level abnormalities in serotoninergic and dopaminergic transporter activities in individuals with ASD [105]. Specifically, they showed a reduced serotonin transporter (SERT) binding capacity in the medial frontal cortex, midbrain, and temporal lobe regions [106]. In contrast, dopamine transporter binding was higher in the orbitofrontal cortex of the autistic group [107].

## 6. Glutamatergic and GABAergic Systems in ASD and the Use of Computational Methods

A disturbance in the equilibrium between excitatory (glutaminergic) and inhibitory (GABAergic) neurons is a hallmark of ASD. The imbalance between the two systems can affect normal brain development and function, altering neurodevelopment and potentially leading to symptoms of autism [108]. An excitatory/inhibitory (E/I) imbalance in autism may result from increased glutamate activity, decreased GABA release, or a reduced number of GABA receptors, explaining the frequent comorbidity and seizures [109,110]. Furthermore, the disruption of synaptic activity between GABA and glutamate may result in neuroinflammation, which is a key cause of ASD [111,112].

The following studies highlight the importance of alterations in the balance between excitatory (glutamatergic) and inhibitory (GABAergic) neurons in autism.

Vattikuti and Chow [113] report that disturbances in a computational model of a local cortical circuit can explain the hypometria (reduced range of motion or gain) and dysmetria (increased performance variability) of saccadic movements reported in ASD studies. Increased stimulation of synapses versus synaptic inhibition results in increased hypometria and dysmetria. This approach may directly link cortical function and ASD behaviors.

Several years later, Deco et al. [114] highlighted that cortical regions exhibit diversity in cytoarchitecture, myeloarchitecture, chemoarchitecture, and interregional connectivity, influencing excitatory to inhibitory cellular activity. These variations affect the balance of inhibition–excitation across brain regions. However, despite the challenges in large-scale models, diverse data now allow for local differences, facilitating a detailed understanding of regional heterogeneity.

The researchers [115] used multiple learning models and large-scale computational models to reveal that changes in cortical networks in autism are associated with dysfunction in the subcortical input to cortical microcircuits and disturbances in excitation/inhibition. The regions involved contain genes expressed in cortical and thalamic areas in early childhood, adolescence, infancy, and young adulthood. The results shed light on the link between microcircuit remodeling in autism and subcortical–cortical connections.

The “hybrid model” of Schirner et al. [116], a new brain network model, contributes to the inference of neurophysiological processes. It can explore hypotheses about emerging phenomena, such as scale-free dynamics and excitation–inhibition balance. It is a form of multimodal fusion, as it uses empirical electroencephalography (EEG) data and tractography connectivity constraints, allowing the connection of structural and functional data.

The research [117] reports that large-scale brain dynamic models, rooted in stochastic calculus, use differential equations for pools of neurons with coupling and stochastic components. These models incorporate nonlinear neuronal dynamics, internal coupling, and noise. Neural mass models (NMMs) and neural field models (NFMs) provide information about fundamental synaptic interactions and biochemical bases (mainly the ionotropic glutamate receptors N-methyl-D-aspartate -AMPA- and α-amino-3-hydroxy-5-methylisoxazole-4-propionic acid -NMDA, as well as GABA). Still, their details can be limited to maintain accessibility and validation. Notably, the ionotropic glutamate receptors NMDA and AMPA enhance synaptic transmission throughout the CNS [118,119]. NMDA hyperfunction and hypofunction [120,121], and reduced AMPA receptor density in cerebellar tissue, have been linked to the autism phenotype [122]. Furthermore, their aberrant activation may contribute to seizure disorders [123], which often coexist with ASD [124].

A few additional studies [125,126] highlight the importance of transmitter receptors in cellular communication in the brain and the response of cells to neurotransmitters, influencing various neurobiological phenomena. Goulas et al. [126] report that positron emission tomography can map the distribution of receptors, enhancing the molecular understanding of brain disorders. They examined the role of receptors in pathologies and drug design, revealing a natural axis of receptor distribution that involves binding to sensory areas, dividing them into ionotropic and metabotropic receptor types, and contributing to controlled predictions.

Reduced GABA and increased glutamate levels link to excitotoxicity and oxidative stress, conditions associated with ASD [127]. According to the research, the altered bacterial community in the intestinal system causes metabolic dysfunction, affecting neurotransmitter and brain function [128]. Specifically, they report the two-way communication between the gut, the central nervous system, and the microbiome [129]. Inadequate intestinal microflora can affect the synthesis and production of neurotransmitters, affecting emotional and cognitive development [130,131]. Mohammad et al. [127] used reconstruction and constraint-based analysis to process given metabolic disorders and quantitatively predict potential biochemical phenotypic states, capturing a metabolic picture of ASD. More specifically, they created a computational framework that contributes to our understanding of gut dysbiosis, predicts oxidative stress, and explores the interactions of GABA and glutamate.

GABA function, influenced by genetics [132], environment, or a combination of factors, is associated with the growth of neuronal connections, which is crucial for optimal network architecture in the adult brain. Early disruptions in GABAergic circuits affect sensory processing, with implications for the developmental process [58].

Hollestein et al. [132] combined the analysis of genetic, structural, and phenotypic data, examining how gene sets associated with inhibitory/excitatory imbalance relate to autism behavioral traits and brain anatomy. Their findings report that glutamate and GABA genes are associated with autism traits and sensory processing, with increased expression in adolescents and adults causing considerable differences in CT scans compared to non-autistic individuals. Suggesting the different roles of genes, which vary during development.

The empirical evidence in humans linking autism to disturbances in excitatory and inhibitory neurotransmission is limited, creating a commonality between animal and human studies. Binocular competition involves two images presented to each eye, with perceptual dominance determined by selective neuronal populations. Perceptual suppression is related to the inhibitory and excitatory dynamics of the cortex. Computational reports suggest that reduced inhibition is associated with autistic perceptual symptoms. Therefore, it may serve as a predictive marker of possible disruption of GABAergic inhibitory signaling in autism [75].

Glutamatergic signaling is crucial in early brain development, regulating neuronal proliferation, differentiation, and synaptic plasticity. It plays a significant role in the pathophysiology of neurodevelopmental disorders, such as intellectual disability and ASD. Imbalances in excitatory/inhibitory synaptic transmission, as a consequence of deficits in synaptic development, signaling, or plasticity, have adverse consequences for cognitive and social development [133]

Freche et al. [134] focused on processes that occur during synaptic transmission and govern long-term plasticity. They reported that perisynaptic microregions modulate synaptic strength and influence signaling output. Their study developed a biophysical computational model to simulate the steps of excitatory synaptic transmission mediated by the AMPA glutamate receptor. The model used Markov chain modeling to account for the receptors’ Brownian motion, the neurotransmitters’ dynamics, and the receptors’ opening and closing. The research found that the presynaptic vesicle release sites are the most sensitive factor in controlling the synaptic current compared to the postsynaptic receptors.

Cortical synapses are dynamic and influenced by presynaptic and postsynaptic cell types. The study of cortical connectivity provides information that enhances our understanding of cognitive abilities. The researchers used microelectrodes to record 1731 synaptic connections in mouse and human neocortexes. Using a synaptic release model, they revealed that excitatory and inhibitory dynamics align with postsynaptic cell subtypes, with synaptic variability driving these differences. However, human excitatory connections showed stability and reliability [135].

A few years earlier, Rosenberg et al. [108] reported that computational models of nonlinear neural circuits can link the genetic and molecular findings about autism with perceptual data. Their study utilized neural network simulations to reveal that divisive normalization (a relationship analysis technique) reduces inhibition, explaining the perceptual consequences of autism and changes in sensory interpretation. This computational perspective helps to understand the genetic basis and behavioral manifestations of autism, providing insights for therapeutic approaches.

Chatterjee et al. [61] created the ASD interrogator (ASDint), a computational model with asymmetric hardware to analyze multiple ASD mechanisms that affect the synapses of glutamatergic neurons. They assessed the variation in synaptic learning parameters in ideal scenario responses and determined the strength and timing of a mechanism’s effect on synaptic function. As a result, the estimation of dysfunctional neurons affecting the coordination of presynaptic and postsynaptic outputs in the neural network improved.

## 7. Computational Approaches’ Contribution to the Identification and Management of ASD

The autism research faces challenges in understanding the complex features of a disorder with intense clinical variability. ASD involves multidimensional processes and marked variations in phenotype compared to other psychiatric disorders. Understanding the early typical versus atypical development of autism spectrum disorder is crucial for the development of effective treatments [16]. The diverse manifestations of the disease make it a complex disorder to diagnose and treat, presenting challenges for individuals in the familiar environment and caregivers. Traditional diagnostic methods rely on behavioral observations and subjective assessments. As a result, they cannot encompass the extent of the severity of its symptoms, requiring more accurate and objective tools [136].

The following highlights the contribution of the collaboration of computational neuroscience with scientific disciplines and the utilization of artificial intelligence tools in the diagnosis and therapeutic approach to autism.

Technology’s development plays a pivotal role in the multidimensional approach and comprehension of ASD. Scientific progress in autism treatment emphasizes the complex interplay between traditional diagnostic tools and artificial intelligence, promoting an inclusive approach that enhances our understanding of the disease [137].

Advances in genetics and neuroscience have reoriented the research from psychosocial to biomedical models, focusing on genetic factors and abnormalities in brain structure and function. Family and twin studies have found a high genetic predisposition to ASD, and interdisciplinary research approaches, including neuroscience, genetics, psychology, and computational modeling, have supported the understanding of the complexity of autism [138].

Computational neuroscience and computational psychiatry collaborate closely. However, the former focuses on developing a mechanistic model of cognitive, emotional, and social processes, emphasizing causal relationships at different brain levels. The latter leverages this knowledge to explore how healthy models can account for deviant behaviors in mental disorders [24]. In addition, computational psychiatry is an expanding field that employs mathematical methods to quantitatively investigate the interacting variables within biobehavioral systems in psychiatric disorders. Using large datasets enhances behavioral and biological diagnostic approaches and helps to subcategorize brain and behavioral dysfunction. It can model neural circuits associated with multifactorial contributions. Therefore, computational methods help predict outcomes for individuals with ASD by describing developmental pathways and identifying critical periods of development that influence functional outcomes [16].

The researchers are using intelligent machine learning methods to improve the diagnosis, processing time, and accuracy of ASD, and reduce data dimensionality for faster access to healthcare, neural networks, support vector machines, decision trees, and rule-based classifiers to create predictive computational models [139]. The systematic review by Moridian et al. [140] highlights the contribution of automated ASD detection to its early prediction. Their study examines autism diagnosis using MRI neuroimaging methods, combining artificial intelligence tools, machine learning, and deep learning.

A similar study by Sherkatghanad et al. [141] developed a convolutional neural network to identify individuals with ASD. They utilized resting-state functional magnetic resonance imaging (fMRI) data from a dataset called the Autism Brain Imaging Exchange (ABIDE). Their model employs fewer parameters than cutting-edge techniques, making it less computationally intensive.

Gao et al. [142] introduced a network that employs multitasking and attentional learning mechanisms to predict and diagnose ASD. This model demonstrated an improved generalization performance and enhanced patient classification capabilities.

Studies have explored machine learning to minimize the need for expert assessments, evaluate observation-based classifier accuracy to expedite autism risk detection, and identify behaviors using feature-specific selection algorithms. Big data and machine learning can analyze clinical categories and common treatment targets across neurodevelopmental trajectories. Having a substantial sample size is crucial. This approach captures the diversity of disorders and helps identify important subgroups. In addition, the computational research enables the comparison of different approaches to predicting outcomes and assessing risk [16].

In a diagnostic study, Rajagopalan et al. [143] analyzed data from the SPARK (Simons Foundation Powering Autism Research for Knowledge) database, including 30,660 participants with and without ASD. The machine learning model, AutMedAI, was validated on independent datasets from SPARK and the Simons Simplex Collection, focusing on phenotypic associations. The model performed well in infancy and early childhood, using minimal background, developmental, and medical information. It also helped identify predictive factors, such as phenotypic behaviors, in detecting the disease.

The researchers are using machine learning methods to diagnose children with autism spectrum disorder who present comorbidities, comparing them with traditional regression models. For example, we mention the study by Song et al. [144], the results of which showed that the machine learning model, specifically the support vector machine (SVM), which includes socio-demographic and behavioral observation data, is effective in identifying the coexistence of ASD with intellectual disability. According to Friston [14], genetic integration is an early stage of computational phenotyping that is effective in classifying or stratifying patients based on their behavioral responses. These genetic models can be used to understand the behavior and interpret their data.

The complex interdependence of autism and the diverse systems require a new paradigm in the systems biology research. The vast amount of information needed for modeling and data analysis makes it imperative to develop new techniques [13]. Neuroimaging studies reveal abnormal brain development trajectories in some children with ASD, disrupting the balance between cellular and synaptic growth and pruning of neurons and synapses [69]. By observing structural and functional differences in certain brain regions and networks, neuroimaging techniques, such as functional magnetic resonance imaging (fMRI), structural magnetic resonance imaging (sMRI), diffusion tensor imaging (DTI), and positron emission tomography (PET), are helping the researchers understand the molecular basis of autism [138].

Positron emission tomography combined with computed tomography (CT) could be a diagnostic tool for identifying autism. F-18 fluorodeoxyglucose positron emission tomography (F-18 FDG PET/CT) is a neuroimaging method that detects functional abnormalities of the whole brain in a short period with applications in ASD [145]. Most patients with autism show significantly low 18FDG uptake on PET imaging, mainly in the hippocampus, amygdala, medial temporal lobe, and cerebellum, and high uptake in the frontal lobes [146].

Ultrasound detection and the measurement of nuchal translucency (NT) are tests conducted during the first trimester of pregnancy to predict chromosomal abnormalities. Hellmuth et al. reported that children with a nuchal translucency over the 99th percentile showed an increased risk of neurodevelopmental disorders, including autism [147]. The study by Kasera et al. utilized a convolutional neural network U-Net to detect the thickening of the nuchal translucency (NT). The researchers used an automated image segmentation algorithm contributing to screening for fetal abnormalities [148].

The study of the connectome, a complete map of neuronal connections in the brain, is supported by AI. The brain’s regions appear as nodes in the network and connections by weighted edges. The researchers use functional magnetic resonance imaging (fMRI) and diffusion magnetic resonance imaging (DMRI) to analyze in vivo connectivity data, with machine learning algorithms crucial for an early autism diagnosis [28]. Μodel-based fMRI uses computational estimates to discover associations between the activity of neuromodulatory nuclei and computational trajectories. It could contribute to creating objective diagnostic tests and developing personalized intervention programs [105,149].

Bioinformatics, a computational biology branch, involves applying in silico tools to understand biological data [150]. The interaction of computational neuroscience and bioinformatics can contribute to the understanding and interconnection of the molecular mechanisms and biophysical determinants involved in neuronal function. Neurons generate electrical signals through dendritic arborizations with ion channels. For the calculation of membrane potential, neurons are small, interconnected cylindrical compartments. These units are represented numerically as electrical circuits. Moreover, precise neuron morphologies are transferred in software settings, such as NEURON and Genesis, for neuronal modeling. Membrane and synaptic properties are distributed through arborization to connect with experimental data or test new hypotheses. However, computational modeling is an exploratory method that requires empirical verification and continuous testing against experimental results to develop new hypotheses and update models [125].

Cognitive computational models also play a significant role, as they identify two essential parameters in interventions: performance on the training set and performance on a generalization set. This distinction is critical in real-world interventions, as it helps to assess whether the intervention generalizes to other subjects or skills. Inductive bias allows computational systems to generate responses to novel inputs. The distinction between training and generalization is also valuable for developmental deficits and interventions focusing on different training methods or generalization performance [151].

Addressing the heterogeneity of autism is crucial for improving diagnostic accuracy and developing targeted cognitive behavioral interventions. However, challenges include increasing sample sizes from diverse cultural and socioeconomic backgrounds, bridging the gap between autism symptomatology and individual differences, and adopting more detailed methods in capturing individual behavioral dynamics [152].

Notable is the use of data science tools to enhance the transformation of ASD interventions. Specifically, machine learning algorithms, natural language processing, network analysis, and predictive analytics facilitate this thorough diagnosis and personalized treatment plans by analyzing patterns in clinical data, behavioral observations, and genetic information. However, applying these methods in the autism spectrum disorder research faces challenges and weaknesses, such as accessibility, scalability, and generalizability. Limited longitudinal studies, ethical concerns, and restricted interdisciplinary collaboration hinder the development of comprehensive patient-centered solutions. Ethical considerations, such as data privacy, algorithmic biases, and potential misinterpretation of machine learning results, pose fundamental factors that need to be addressed [136].

More generally, the rapid evolution of technology has improved health, information management, productivity, safety, and knowledge delivery, but has also highlighted weaknesses in data management, excessive expectations, and reliability issues [153]. As indicated, to promote fair and impartial care for each individual is necessary to ensure diversity in ASD datasets. By embracing this diversity, we can pave the way for transformative developments in healthcare [154]. Furthermore, by incorporating technology and artificial intelligence in handling ASD symptoms, we shape fair and inclusive conditions for all [155]. Table 2 gathers all the major findings regarding the dysfunction of key neurotransmitters (serotonin, dopamine, glutamate, and GABA) in ASD and the computational neuroscience methods that were used.

## 8. Discussion

Recent discoveries in neuroscience and artificial intelligence have made remarkable strides due to the visionary pioneers who laid the foundations in both fields. However, the thriving interaction between them has diminished, overshadowed by the increasing complexity of each discipline and the blurring of academic boundaries [156].

Computational neuroscience is an emerging scientific discipline focusing on how the brain processes information through experimental, analytical, and modeling methods. It involves designing computational frameworks to examine the properties of neural systems at different levels of detail, often including the simulation of numerical models on computers and experimental verification [71].

The resulting functional outcome of brain activation, including memory formation, thoughts, and emotions, is difficult to quantify due to the complexity of the data and the difficulty in uncovering mechanistic connections between neural processes. Most causal hypotheses are correlational, implying that changes in behavior or neurological function are associated with changes in brain electrical activity or structure. Consequently, collecting more information was expected to enhance the interpretation of the data. However, animal models do not lead to causal conclusions and often do not improve the explanation of the information collected [6].

Computational models fill deficiencies in the research process by helping us understand complex phenomena by exploring the causal pathways from variable changes to their effects on other variables. They employ different levels of analysis to illustrate the interdependence and influence of these variables, leading to a comprehensive understanding of the phenomenon under investigation [157]. Furthermore, computational frameworks leverage diverse neuroscientific data gathered from multiple studies that focus on specific parameters. This combination of evidence includes online, controlled laboratory data, and neuroimaging data. Understanding neurobiological mechanisms through the systematic research is crucial for advancing personalized mental health using neuroscience. Furthermore, a polytropic mindset encourages the integration of new neuroimaging and behavioral methods [158].

According to Law et al. [159], whole-brain modeling allows for the methodical manipulation of brain structure and function. Furthermore, by examining various consequences and linking system-level dynamics to molecular substrates, the fragmentation within the neuroscience field is reduced by conveying information at multiple levels. However, models are often constrained by the vast number of variables that influence the structure and function of the nervous system, frequently accounting for only specific biological variables. A comprehensive model must be sufficiently simple to produce clear findings at the highest level to achieve overall efficacy [6].

Autism is a neurological disorder marked by increased brain connectivity, neuronal migration deficits, excitatory/inhibitory imbalance, and synaptic dysregulation. Genetic heterogeneity is apparent, with genes associated with ASD interacting with neurodevelopmental pathways. Detecting structural and functional change requires non-invasive imaging techniques, such as magnetic resonance imaging, which reveals altered cortical structures and reduced white matter connectivity. Models utilizing artificial intelligence and neuroimaging data enhance the accurate diagnosis and identification of early diagnostic biomarkers in ASD. In addition, genetic neuroimaging approaches improve the understanding of alterations in the neurochemical system and molecular pathways related to autism. In particular, neuroimaging genetics effectively examines the effects of genetic variants on brain connectivity and function [149].

The research has shown that applying computational models during sensitive developmental periods reveals considerable information about the evolution of functional plasticity, contributing to our understanding of the interacting factors that limit neuroplasticity [76]. Neurotransmitters are vital for transmitting information within the CNS and peripheral nervous system, affecting various functions, such as emotions, thoughts, and sleep. Imbalances in neurotransmitter levels can lead to brain function dysregulation, resulting in physical, psychotic, and neurodegenerative diseases [160].

Real-time detection of neurotransmitters is an exciting frontier in neuroscience. NTs create complex concentration gradients between neurons, bind to receptors, and trigger a cascade of biochemical responses that support our brain function. However, accurately quantifying these dynamic molecules remains challenging, given their rapid release and action in the synaptic environment [161].

A prominent neurobiological disorder in autism is the disturbed development of multiple neurotransmitter systems, emphasizing the glutamate, GABAergic, and serotonergic systems. The declining synaptic ratio of excitation to inhibition is attributed to increased or decreased glutamatergic or GABAergic signaling [162].

Moreover, the role of dopaminergic signaling in the manifestation of autism symptoms is emerging as essential. Computational studies report the ability of DA signaling to modify ionic and synaptic currents and enhance GABA release in the prefrontal cortex (PFC). Impaired dopaminergic signaling is associated with numerous brain disorders due to its critical role in various brain processes, cognitive functions, and behavioral control. An examination of the clinical features of DA signaling can reveal evidence of dopaminergic modulation in the brain, which can cause neurological and psychiatric disorders. Furthermore, computational models can simulate the role of DA in neuropsychiatric disorders, potentially improving treatment techniques by testing different DA agonists or antagonists without human intervention [95].

Understanding the chemical diversity and complexity of the brainstem chemoarchitecture is crucial for interpreting and predicting the response to therapeutic interventions targeting serotonergic and other neurotransmitter systems in autism [163,164]. The research suggests that the intense interactions between neurotransmitter systems pose a challenge for identifying composite elements in observed data. Combining biophysical models with an information processing perspective can help address the methodological challenges by understanding the computational variables or processes encoded or activated by neurotransmitters [165,166].

A thorough examination of neuroscience data advances computational modeling and can provide insights for modeling intact and impaired cognitive abilities. Progress in computational models of developmental disorders provides the basis for intervention exploration. However, questions remain about the ability to bridge the gap between theoretical approaches and treatment programs with a well-founded computational foundation [151]. The ability of models to combine data from different studies is highlighted, providing a concise description of phenomena, revealing underlying mechanisms, and predicting new experimental procedures and theories [167]. Hauser et al. [29] argue that the unification of mechanism-driven models and their performance has a lot of potential for creating biologically informed and transparent predictive models, enabling the creation of innovative therapeutic approaches and effective interventions.

In summary, computational neuroscience combines elements of cognitive science, psychiatry, artificial intelligence, and other scientific disciplines to understand brain activity and the nervous system. Harnessing computational approaches significantly enhances our understanding of behavioral patterns and cognitive mechanisms in autism, paving the way for more accurate diagnoses and innovative intervention strategies. By studying neurotransmitters, genetic factors, and alterations in neural networks, computational neuroscience contributes to creating personalized therapies.

### Limitations and Suggestions for Future Research

The increasing incidence of ASD symptoms in children worldwide has created the need to search for computational methods with capabilities for analyzing and processing complex data to address the persistent impairments of the disorder. The present study focused on connecting computational neuroscience with the understanding of neurotransmission in autism, summarizing analyses and simulations of neuronal systems, particularly serotonergic, dopaminergic, glutamatergic, and GABAergic systems, aiming to understand the neurobiological mechanisms better. However, this study did not include demographic characteristics (gender and age), motivating further studies.

A significant limitation of this study is the lack of reference to modern methods that contribute to the understanding of the function of ion channels and neurotransmission in the brain.

Understanding the relationship between E/I imbalance and structural differences in autism is crucial for understanding its molecular and genetic mechanisms. Modern in-depth research could contribute subtype markers and targeted therapeutic options.

Computational modeling simulations that provide comprehensive analyses and data on changes in synaptic function could improve our understanding of the neuropathology of autism, which is a limitation of the research. In particular, a targeted study to document structural changes in synaptic plasticity, including receptors and biochemical signaling, would contribute to understanding synaptic mechanisms, providing the potential for more effective therapeutic approaches.

The difficulty of identifying and understanding etiological factors that compose the heterogeneous condition of ASD is a major limiting factor in the adoption of effective interventions. Utilizing machine learning and big data on a large population sample can significantly enhance the investigation, analysis, and comparison of biological factors. Additionally, this approach can aid in defining new subgroups with shared characteristics, thereby facilitating the development of tailored therapeutic methods.

## 9. Conclusions

Computational neuroscience harnesses the power of cognitive science, computational psychiatry, mathematics, computer science, artificial intelligence [168], and other fields to uncover the complexities of brain activity and the nervous system. This innovative approach not only enhances the diagnosis and treatment of autism but may also uncover genetic factors associated with this condition through the latest advances in artificial intelligence.

Neurotransmitters are chemical messengers in brain cells that affect brain chemistry and our perception of the world. In addition, their influence on brain structure and development has significant implications for the manifestation of ASD symptoms. Computational neuroscience delves into the interaction between neurotransmitters and the human nervous system, harnessing the power of machine learning and neural network models. These advanced models, utilizing mathematical analysis and algorithmic correlations, simulate the brain’s complex processes. They highlight the critical role of neurotransmitters in enhancing synaptic conduction and facilitating signal transmission. Genes related to neurotransmitter metabolism influence their function and the manifestation of autism. Significant correlations of dopaminergic activity in the striatum and prefrontal cortex impact the executive mechanism, highlighting in parallel the dominant role of the basal ganglia in dopaminergic gating. The effect of synaptic interactions is associated with the biochemical bases of neurotransmitter receptors and intestinal dysbiosis. It is also significant that the disturbance of excitatory-inhibitory signaling involves genetic factors and alterations in brain networks, affecting the pathophysiology of autism.

Computational approaches are revolutionizing the diagnostic process by enhancing efficiency, optimizing data dimensionality and diagnostic time, and revealing key behaviors. They are effective for categorizing autism disorders, comparing treatment strategies, and supporting personalized interventions. They reveal structural and functional differences in brain activity, facilitating training and evaluating the generalizability of interventions. However, the effectiveness of computational modeling depends on key factors, such as reliability, validity, validation, and generalizability.

## Figures and Tables

**Table 1:** Neurotransmitter types, mechanisms, and functions.

Neurotransmitter	Type	Functions	Associated Conditions	Pathways/Mechanisms
Serotonin (5-HT) [35,36,37,38,39,40]	Inhibitory, Modulatory	Regulates feeding, sleep, aggression, emotion, body weight, circadian rhythm, reward processing, and plasticity	OCD, anxiety, alcoholism, emotional disorders, ASD, memory, and learning deficits	Produced in dorsal and median raphe nuclei; modulates glutamate and GABA transmission; acts via multiple receptors; impacts cortical synaptic plasticity and morphogenesis
Dopamine (DA) [17,41]	Modulatory, Excitatory	Influences behavior, reward processing, emotional regulation, and cognition	ASD, Parkinson’s, emotional dysregulation, aggression, and prolactin dysregulation	Four pathways: Nigrostriatal, Mesolimbic, Mesocortical, and Tuberoinfundibular; overactivity leads to impulsivity and underactivity leads to cognitive deficits
Glutamate (Glu) [31,41,46,47,48,49]	Excitatory	Enhances memory and promotes synaptic excitation across brain	ASD, seizures, hypersensitivity, oxidative stress, and sensory/memory/emotional dysfunctions	Synthesized from glutamine; converted in astrocytes; interacts with GABA system; involved in excitatory neurotransmission across nearly all synapses
GABA [42,43,44,45,46,47,48,49]	Inhibitory	Regulates cell proliferation, migration, maturation, and death	ASD, information processing challenges, social skill development deficits, seizures, anxiety, and repetitive behaviors	Main inhibitory neurotransmitter; interacts with glutamate; part of GABAergic system; critical to neurodevelopment and neurotransmission balance

**Table 2:** Dysfunction of neurotransmitters in ASD and how they are studied via computational neuroscience.

Neurotransmitter	Dysfunction in ASD	Computational Methods
Serotonin (5-HT) [36,38,82,83,84,85,86,89,90]	Altered serotogenic signaling; hyperseretonemia in 25–33% of ASD patients; linked to aggression, hyperactivity, sleep, and sensory issues	Mathematical models of serotonergic homeostasis; gut-brain axis modeling; PET data provide information about serotonin function; fMRI classifiers model growth and spatial distribution of axon density
Dopamine (DA) [50,94,95,96,97,98,99,100,101,102,103,104,105,106,107]	Abnormal DA signaling affects reward processing, motivation, repetitive behaviors, and executive functions	Reinforcement learning (TDRL); cross-task models (XT); biophysical models; genetic simulations of DA transport (DAT); fMRI and PET with computational models
Glutamate (Glu) [108,117,118,119,120,121,122,123,124,134]	Excitatory/inhibitory imbalance; hyperactive NMDA/AMPA receptors; associated with seizures and oxidative stress	Biophysical models of AMPA receptor activity; synaptic learning parameter models; neural field models (NFMs); structural synapse simulations
GABA [75,108,109,110,111,112,113,114,115,116,126,127,128,129,130,131,132]	Reduced GABAergic activity; decreased inhibition leads to E/I imbalance and sensory/perceptual abnormalities	Neural circuit simulations; binocular competition modeling; hybrid EEG models; gut microbiome interaction modeling
